# The ErbB Signaling Network and Its Potential Role in Endometrial Cancer

**DOI:** 10.3390/epigenomes7040024

**Published:** 2023-10-01

**Authors:** Georgios Androutsopoulos, Ioanna Styliara, Evgenia Zarogianni, Nadia Lazurko, George Valasoulis, Georgios Michail, Georgios Adonakis

**Affiliations:** 1 Gynaecological Oncology Unit, Department of Obstetrics and Gynaecology, School of Medicine, University of Patras, 26504 Rion, Greece; 2 Department of Obstetrics and Gynaecology, School of Medicine, University of Patras, 26504 Rion, Greece; anni.styl@gmail.com (I.S.); tzenizar91@gmail.com (E.Z.); nadialazurko@gmail.gr (N.L.); gmichail@upatras.gr (G.M.); adonakis@upatras.gr (G.A.); 3 Department of Obstetrics and Gynaecology, Medical School, University of Thessaly, 41334 Larisa, Greece; gvalasoulis@gmail.com; 4 Hellenic National Public Health Organization—ECDC, 15123 Athens, Greece

**Keywords:** ErbB receptors, EGF system, physiology, signaling pathways, carcinogenesis, expression profile, clinical role, endometrial cancer

## Abstract

Endometrial cancer (EC) is the second most common malignancy of the female reproductive system worldwide. The updated EC classification emphasizes the significant role of various signaling pathways such as PIK3CA-PIK3R1-PTEN and RTK/RAS/β-catenin in EC pathogenesis. Some of these pathways are part of the EGF system signaling network, which becomes hyperactivated by various mechanisms and participates in cancer pathogenesis. In EC, the expression of ErbB receptors is significantly different, compared with the premenopausal and postmenopausal endometrium, mainly because of the increased transcriptional activity of ErbB encoding genes in EC cells. Moreover, there are some differences in ErbB-2 receptor profile among EC subgroups that could be explained by the alterations in pathophysiology and clinical behavior of various EC histologic subtypes. The fact that ErbB-2 receptor expression is more common in aggressive EC histologic subtypes (papillary serous and clear cell) could indicate a future role of ErbB-targeted therapies in well-defined EC subgroups with overexpression of ErbB receptors.

## 1. Introduction

Endometrial cancer (EC) is the second most common malignancy of the female reproductive system worldwide [1]. It is more prevalent in wealthy and more developed regions (North America, Europe, Australia, New Zealand), compared with less developed ones (Central and South America, Asia, Africa) [1]. However, mortality rates are considerably higher in less developed areas (northern Africa, Melanesia) [2,3]. The disease usually affects postmenopausal women, with an average annual incidence reaching 2.1% [1]. Although the vast majority of EC patients are postmenopausal, approximately 14% of them are premenopausal and almost 4% are below 40 years of age [4,5,6,7,8,9,10,11,12,13,14,15,16,17,18,19,20,21,22].

Current evidence does not support any screening methodology for early detection of EC, as cervical cytology performs poorly [23]. However, the fluorescence in situ hybridization test (FISH) in vaginal swab specimens has shown promising results in EC detection [24]. Artificial intelligence and machine learning algorithms have been proposed to assist in the discrimination between benign and malignant endometrial nuclei, obtained via image analysis and measured from liquid-based cytology slides and lesions so far. These show promising results, as their performance appears to be similar to that of traditional regression models in EC [25,26,27,28]. Moreover, biospectroscopy has several applications in biomedical science, from detecting toxins and pollutants in the human body to identifying areas of stem cells in human tissue. It can effectively identify biomarkers of disease states at many organ sites without the need for staining or isotopic labeling [29,30]. Apart from that, pelvic ultrasound scans or saline infusion sonograms can be offered on a 1–2-yearly basis in the context of routine gynecological examination, except for individuals undergoing close follow-up for hereditary, non-polyposis colon cancer (HNPCC) [31].

In the past, the sporadic classification of EC cases was based on clinical, metabolic, endocrine and pathological features [32,33]. More recently, genomic data including somatic mutation rates, frequency of copy number alterations and MSI status have been used to create an updated EC classification, reflecting the increased impact of molecular biology in disease progression and patients’ outcome [34,35]. Moreover, the updated EC classification emphasizes the significant role of various signaling pathways, such as PIK3CA-PIK3R1-PTEN and RTK/RAS/β-catenin, in EC pathogenesis [34,35,36].

Some of these pathways are part of the EGF system signaling network, which becomes hyperactivated with various mechanisms (gain of function mutations, genomic amplification, chromosomal rearrangements and autocrine activation) and participates in cancer pathogenesis [37,38,39,40,41,42].

Our aim is to provide an update on current knowledge of the signaling network of ErbB receptors and their participation in cancer pathogenesis, as well as their potential clinical role in EC cases.

## 2. Physiology of ErbB Receptors 

The EGF system is present in various human organs and plays a significant role in cell proliferation, differentiation, migration and apoptosis during embryogenesis and postnatal development [39,43,44].

### 2.1. ErbB Receptors

ErbB receptors are members of the subclass I superfamily of receptor tyrosine kinases (RTKs) [37,39,45]. In humans, the EGF system consists of the following ErbB receptors: epidermal growth factor receptor (EGFR), ErbB-2, ErbB-3 and ErbB-4 [37,39,44,45,46]. These receptors are trans-membrane glycoproteins that catalyze the transferring of γ phosphate of ATP to hydroxyl groups of tyrosines in target proteins [47]. However, ErbB-3 has no intrinsic tyrosine kinase activity, and it depends on another ErbB receptor (usually ErbB-2) for intracellular signaling [45,48].

**Figure 1:**
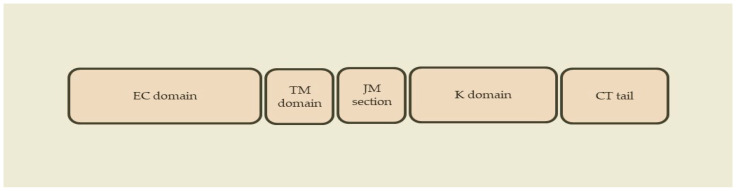
Schematic structure of ErbB receptors. EC domain: extracellular ligand-binding domain. TM domain: transmembrane domain. JM section: juxtamembrane section. K domain: intracellular bilobed tyrosine kinase domain. CT tail: tyrosine-containing C-terminal tail.

Regarding their structure, ErbB receptors have an extracellular ligand-binding domain, a transmembrane domain, a short juxtamembrane section, an intracellular bilobed tyrosine kinase domain and a tyrosine-containing C-terminal tail (Figure 1) [44,45,46,49].

The extracellular ligand-binding domain is divided into four subdomains: L1 (or I), CR1 (or II), L2 (or III) and CR2 (or IV) [44,46,49]. The leucine-rich subdomains L1 and L2 participate in ligand binding [44,46,49]. The cysteine-rich subdomains CR1 and CR2 participate in disulfide bond formation, while subdomain CR1 contains a β-hairpin loop and participates in ErbB receptors’ homodimerization and heterodimerization [44,46,49]. Moreover, the intracellular tyrosine kinase domain is subdivided into two lobes, N and C [44,46].

### 2.2. ErbB Ligands

In humans, the EGF system has the following ErbB peptide mediators (ligands): EGF, transforming growth factor-a (TGF-a), amphiregulin (AR), heparin-binding growth factor (HB-EGF), betacellulin (BTC), epigen, epiregulin (EPR), neuregulin-1 (NRG-1), neuregulin-2 (NRG-2), neuregulin-3 (NRG-3), neuregulin-4 (NRG-4), neuroglycan C and tomoregulin [39,44,45,46]. Ligand binding to the extracellular domain of the ErbB receptor results in conformational changes and induces homodimerization and heterodimerization of receptors [37,44,45,46]. However, the ErbB-2 receptor fails to bind any ligands [37,44,45,46].

Based on their affinity for one or more receptors, ErbB ligands could be further classified into the following subgroups:

1. Ligands with binding specificity for EGFR only: EGF, TGF-a and AR [44,45,46].

2. Ligands with dual binding specificity for EGFR and ErbB4: HB-EGF, BTC and EPR [44,45,46].

3. Ligands with binding specificity for ErbB-3 only: neuroglycan C [44,45,46].

4. Ligands with binding specificity for ErbB-4 only: NRG-3, NRG-4 and tomoregulin [44,45,46].

5. Ligands with dual binding specificity for ErbB-3 and ErbB-4: NRG-1, NRG-2 [44,45,46].

All these data are presented in detail in Table 1.

It should be emphasized that ErbB ligands usually act a short distance from the cells producing them [46,50]. Overall, ErbB ligands may act either on the same cell (autocrine signaling), on an adjacent cell (juxtacrine signaling) or on a nearby cell (paracrine signaling) [46,50].

### 2.3. Receptor Homodimerization and Heterodimerization

There are two distinct conformations of the extracellular ligand-binding domain, based on the activation status of EGFR, ErbB-3 and ErbB-4 receptors:

1. Closed conformation. When ErbB receptors are inactive, there are intramolecular interactions between the cysteine-rich subdomains CR1 and CR2, causing closed conformation of the extracellular ligand-binding domain [44,45,46,51,52].

2. Open conformation. When ErbB receptors become active, the leucine-rich subdomains L1 and L2 create a ligand-binding pocket, allowing interactions with a single ligand, while the extracellular ligand-binding domain takes an open conformation and the β-hairpin loop dimerization arm of subdomain CR1 is exposed [44,45,46,51,52].

It seems that there is equilibrium between both conformations of the extracellular ligand-binding domain, related directly to ligand presence and subsequent ligand binding [51,52,53]. More specifically, ligand binding to the leucine-rich subdomains L1 and L2 stabilises the extracellular ligand-binding domain to an open conformation, exposes the β-hairpin loop dimerization arm of subdomain CR1 and allows receptor homodimerization and heterodimerization [44,45,46,52,53,54]. Subsequently, ErbB receptor dimerization induces conformational changes of the intracellular bilobed tyrosine kinase domain [44,45,46,55,56].

In contrast, the extracellular ligand-binding domain of the ErbB-2 receptor has an extended conformation that is not suitable for ligand binding, as there is close proximity of the leucine-rich subdomains L1 and L2, abolishing the ligand-binding site [44,45,46,57,58,59]. However, the extended conformation of the ErbB-2 receptor is necessary for interaction with other ErbB receptors and subsequent ligand-independent heterodimerization and signaling [44,45,46,57,58,59]. Moreover, abnormal overexpression of the ErbB-2 receptor permits ligand-independent receptor homodimerization [44,46,58].

Overall, homodimerization and heterodimerization of ErbB receptors represents an essential part in the pathophysiology of the EGF system signaling network [44,45,46,55,56]. Furthermore, the ErbB-2 and ErbB-3 heterodimer is the most transforming and mitogenic receptor complex [60].

### 2.4. Intracellular Tyrosine Kinase Activation

Following homodimerization and heterodimerization of ErbB receptors, conformational changes of the intracellular tyrosine kinase domain take place, which in turn cause tyrosine kinase activation and phosphorylation of the tyrosine-containing C-terminal tail [44,45,46,55,56].

As already mentioned, the intracellular tyrosine kinase domain has a bilobed structure, with ATP binding between the N and C lobes [44,45,46,56]. More specifically, the C-lobe of an intracellular tyrosine kinase domain (activator) allosterically interacts with the N-lobe of another intracellular tyrosine kinase domain (receiver) within the same dimerization pair [44,45,46,56]. This interaction induces conformational changes in the N-lobe of the receiver tyrosine kinase and finally causes its activation [44,45,46,56]. Subsequently, the activated receiver tyrosine kinase catalyzes phosphorylation of tyrosine residues in the tyrosine-containing C-terminal tail of the activator tyrosine kinase [44,45,46,56]. These phosphorylated tyrosine residues serve as docking sites for adaptor proteins, enzymes and various signaling molecules containing Src homology 2 (SH2) and phosphotyrosine binding (PTB) domains [38,44,45,46,56,61,62].

## 3. Signaling Pathways

**Figure 2:**
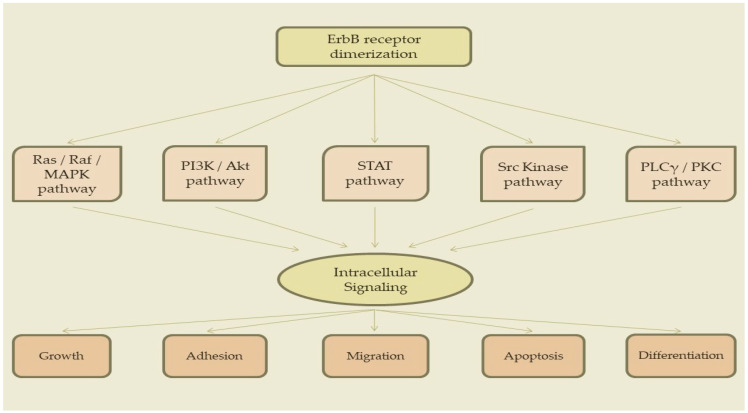
ErbB receptors’ signaling pathways.

Activation and subsequent autophosphorylation of ErbB receptors enables recruitment of various signaling molecules containing the SH2 and PTB domains, that result in downstream signaling via several pathways (Figure 2) [38,42,44,45,46,56,61,62,63]:

### 3.1. Ras/Raf/MAPK Pathway

The Ras/Raf/mitogen-activated protein kinase (MAPK) pathway has a fundamental role in cell biology, mainly as a transducer of extracellular signals to cellular responses [63,64]. Ιt is actively involved in cell cycle regulation (proliferation, differentiation, migration and apoptosis), integrin signaling, tissue repair and angiogenesis [64,65,66,67].

Following ErbB receptor activation and phosphorylation of the tyrosine-containing C-terminal tail, the activated ErbB receptor recruits, directly or indirectly (through the Shc adaptor protein), an adaptor protein named growth factor receptor binding protein 2 (Grb2) via its SH2 domain (Src homology 2) [64,68,69,70,71,72]. Subsequently, the SH3 domain of Grb2 interacts with the proline-rich C-terminal domain of Son of Sevenless (Sos) in order to create an ErbB receptor–Grb2–Sos complex [64,68,71,72]. This leads to Sos translocation to the cell membrane and enables its interaction with Ras [72,73,74].

The interaction between Sos and Ras causes conformational changes and allosteric activation of Sos through a rotation of its REM domain [72,73,74,75]. The allosteric activation of Sos allows Ras binding and promotes replacement of GDP with GTP in Ras that leads to Ras activation (Ras-GTP) and initiation of the Ras pathway [72,73,75,76].

More specifically, Ras–GTP recruits and dimerizes Raf-1 protein kinase on the inner side of the cell membrane, in order to activate it through tyrosine phosphorylation [72,77,78]. Subsequently, activated Raf-1 interacts and activates MAPK/ERK kinase (MEK1 and MEK2), which in turn phosphorylates, activates and anchors to the cytoplasm downstream proteins such as extracellular signal-regulated kinases (ERK1 and ERK2) [64,72]. Then, activated ERK1 and ERK2 translocate to the nucleus in order to phosphorylate and activate various nuclear transcription factors involved in cell proliferation, differentiation and migration [63,64,72].

Overall, the Ras/Raf/MAPK pathway is implicated in a wide variety of cellular biological functions, but is also related to tumorigenesis [64,72].

### 3.2. PI3K/Akt Pathway

The phosphatidylinositol 3-kinase (PI3K)/Akt pathway has an essential role in cell biology, mainly in transduction of extracellular signals to intracellular messages [79]. Ιt is actively involved in cell cycle regulation (proliferation, migration and apoptosis) and cytoskeletal rearrangement [80].

Following ErbB receptor activation and phosphorylation of the tyrosine-containing C-terminal tail, the activated ErbB receptor directly recruits the PI3K (subclass IA) regulatory subunit via its SH2 domain and causes allosteric activation of the PI3K catalytic subunit [79,81,82]. Subsequently, activated PI3K catalyzes conversion of phosphatidylinositol (4, 5) bisphosphate (PIP2) to phosphatidylinositol (3, 4, 5) trisphosphate (PIP3) at the cell membrane [79,81]. Then PIP3 provides docking sites for signaling proteins with pleckstrin homology (PH) domains, including 3-phosphoinositide-dependent kinase 1 (PDK1) and serine-threonine protein kinase Akt (protein kinase B (PKB)) [79,81,82].

In particular, PIP3 directly recruits Akt to the cell membrane via its PH domain and this results in Akt conformational changes and exposure of two crucial amino-acid residues (Thr308 and Ser473) [79,80,83]. Thr308 is phosphorylated by PDK1, while Ser473 is phosphorylated by PDK2 [79,80,81,83,84]. Both phosphorylation events are necessary for full Akt activation, which in turn phosphorylates many cytoplasmic and nuclear proteins and regulates a wide range of cellular processes involved in protein synthesis, cell cycle progression and cell survival [79,80,81,82,83,84].

It is interesting to note that specific docking sites for the PI3K (subclass IA) regulatory subunit are present on the ErbB-3 receptor, while they are absent on the EGFR receptor [63,85]. Moreover, EGFR-dependent PI3K activation occurs either through EGFR and ErbB-3 dimerization or through a Gab-1 docking protein [63,86].

Overall, the PI3K/Akt pathway is implicated in various cellular processes and plays an important role in carcinogenesis [63,80].

### 3.3. STAT Pathway

The signal transducers and activators of transcription (STAT) pathway has a principal role in cell biology, mainly as a transducer of extracellular cytokine signals to cellular responses [87,88,89]. Ιt is actively involved in cell cycle regulation (proliferation, differentiation, migration and apoptosis) [87,88,89].

Following ErbB receptor activation and phosphorylation of the tyrosine-containing C-terminal tail, the activated ErbB receptor can cause JAK—independent tyrosine phosphorylation of STAT proteins, probably via the Src kinase [87,88,89,90]. Phosphorylated and activated STAT proteins create dimers via SH2 domain interactions and translocate to the nucleus, where they bind to specific DNA sequences in gene promoters and regulate gene transcription [87,88,89,91].

Overall, the STAT pathway is implicated in various developmental and homeostatic processes, but also related to tumorigenesis [87,88,89,91].

### 3.4. Src Kinase Pathway

The Src kinase pathway has a critical role in cell biology, especially as a transducer of extracellular signals to cellular responses [92]. Ιt is actively involved in cell cycle regulation (proliferation, adhesion, migration and apoptosis), integrin signaling and angiogenesis [91,92].

Following ErbB receptor activation and phosphorylation of the tyrosine-containing C-terminal tail, the activated ErbB receptor recruits Src kinase via its SH2 domain and causes Src activation [91,93]. Subsequently, activated Src acts as signal transducer and enhancer of ErbB receptor activation [63,94,95].

More specifically, Src activates many downstream proteins (p130^Cas^, FAK, PI3K, VEGF, HIF1α and STAT) through tyrosine phosphorylation [91]. Furthermore, Src regulates various cell cycle proteins (c-Myc, cyclin D and p21) through transcriptional and post-translational mechanisms [91].

Overall, the Src kinase pathway is implicated in many cellular processes and plays an important role in carcinogenesis [63,91,96,97].

### 3.5. PLCγ/PKC Pathway

The phospholipase Cγ (PLCγ)/protein kinase C (PKC) pathway has an essential role in cell biology, mainly in transduction of extracellular signals to intracellular messages [46,98]. Ιt is actively involved in cell cycle regulation (proliferation, differentiation and migration) and angiogenesis [46,98].

Following ErbB receptor activation and phosphorylation of the tyrosine-containing C-terminal tail, the activated ErbB receptor recruits PLCγ via its SH2 domain and causes PLCγ phosphorylation and activation [98,99,100]. Subsequently, activated PLCγ catalyzes hydrolysis of phosphatidylinositol (4, 5) bisphosphate (PIP2) to inositol (1, 4, 5) trisphosphate (IP3) and (1, 2) diacylglycerol (DAG) [95,98,101]. IP3 has significant role in intracellular calcium release, while DAG is cofactor in protein kinase C (PKC) activation [46,63,95]. Activated PKC catalyzes phosphorylation and activation of several transcription factors [46]. Moreover, PKC is actively involved in multiple signaling components, including MAPK and JNK pathways [46,63,95,102,103].

Overall, the PLCγ/PKC pathway is implicated in many cellular processes and plays an important role in carcinogenesis [46,98].

## 4. Epigenetic Regulation of ErbB Signaling

As already mentioned, the activation and subsequent autophosphorylation of ErbB receptors enables recruitment of various signaling molecules and results in downstream signaling via several pathways [38,42,44,45,46,56,61,62,63].

However, heritable changes in gene function without alterations in the DNA sequence (epigenetic changes) could possibly affect ErbB—mediated signal transduction and gene transcription via several mechanisms [104,105]:

### 4.1. DNA Methylation

DNA methylation is an extensively studied mechanism of epigenetic alterations [106]. DNA methylation patterns (methylation and demethylation) are regulated by specific enzymes and subsequently affect gene transcription [106,107].

More specifically, DNA methylation is catalyzed by the family of DNA methyltransferase (DNMT) enzymes, which transfer methyl groups from S-adenosyl-L-methionine (SAM) to cytosine residues and form 5-methylcytosine (5-mC) [106,108]. The majority of DNA methylation occurs in CpG islands, in which cytosine is followed by a guanine [106]. Most CpG islands are present in promoters and their methylation leads to transcriptional silencing [106,108]. Especially in ErbB signaling, PTEN promoter hypermethylation suppresses PTEN expression and activity, with a direct effect on PI3K/Akt pathway signaling [104,109].

Likewise, DNA demethylation is achieved either by active enzymatic demethylation or by passive replication—dependent on the dilution of methylation [108]. Particularly in active enzymatic demethylation, 5-mC undergoes a series of oxidation reactions catalyzed by the methylcytosine dioxygenases Ten-Eleven-Translocation (TET) enzymes [108,110]. The 5-hydroxymethylcytosine (5hmC) is the first intermediate of active DNA demethylation [108]. Enrichment of 5hmc in promoter regions is often associated with activation of gene expression [108]. In this way, DNA demethylation links to genomic instability [106,108]. Especially in ErbB signaling, Ras promoter hypomethylation enhances Ras expression and activity, with a direct effect on signaling of the Ras/Raf/MAPK and PI3K/Akt pathways [104].

### 4.2. Histone Modification

Histone modifications represent another mechanism of epigenetic alterations [106]. They affect lysine and arginine residues on histone tails, which are targets of covalent post-transcriptional modifications (acetylation, methylation, phosphorylation and ubiquitylation) [106,108].

More specifically, histone acetylation occurs through the addition of an acetyl group to the lysine residues in histone tails [106]. Histone acetyltransferases (HATs) add acetyl groups and are associated with active gene transcription at promoter and enhancer sites [106]. In contrast, histone deacetylases (HDACs) remove acetyl groups and are associated with gene silencing and transcriptional repression [106]. Especially in ErbB signaling, EGFR acetylation by CREB-binding protein (CBP) acetyltransferase affects receptor phosphorylation and subsequent activation [104,111].

Likewise, histone methylation occurs through the addition of methyl groups to the arginine or lysine residues in histone tails [106]. Histone methyltransferases (HMTs) add methyl groups and are associated with both active gene transcription and gene repression [106]. In contrast, histone demethylases (HDMs) remove methyl groups [106].

### 4.3. Non-Coding RNAs

Non-coding RNAs (ncRNAs) are functional RNA molecules that occupy a large fraction of the genome, but they are not translated into proteins [108,112,113,114]. They have key roles in the regulation of gene expression at transcriptional and translational levels [113,114]. Moreover, they can be divided into the following categories: microRNAs (miRNAs), long noncoding RNAs (lncRNAs), small interfering RNAs (siRNAs), small nuclear RNAs (snRNAs), small nucleolar RNAs (snoRNAs), ribosomal RNAs (rRNAs), transfer RNAs (tRNAs), circular RNAs (circRNAs) and PIWI-interacting RNAs (piRNAs) [108,112,113,114]. Among these, miRNAs and lncRNAs have crucial roles in cancer epigenetics [113,114].

More specifically, miRNAs are small ncRNAs, approximately 19 to 22 nucleotides in length, that regulate gene expression by posttranscriptional silencing [115,116]. They usually bind to the 3′-untranslated region (3′-UTR) of target messenger RNA (mRNA) molecules, resulting in either translational inhibition or mRNA degradation [113,114,115,117].

In contrast, lncRNAs are larger ncRNAs, more than 200 nucleotides in length, that regulate gene expression at transcriptional, post-transcriptional and epigenetic levels [113,118,119]. In particular, guide lncRNAs act by recruiting or rejecting epigenetic regulators (chromatin modifying complexes and chromatin remodeling complexes) onto specific chromosomal loci [120]. Architect lncRNAs act by modifying the three-dimensional chromatin conformation [120]. Enhancer lncRNAs regulate gene transcription through enhancer-like functions [120]. Moreover, lncRNAs regulate DNA methylation status by recruiting or inhibiting DNA methyltransferases and demethylases [120]. Furthermore, lncRNAs regulate mRNA stability, protein—protein interactions and post-translational protein modifications [113,121,122,123,124].

## 5. EGF Dysregulation and Carcinogenesis

Dysregulation of the EGF system signaling network participates in the pathogenesis of various diseases (diabetes, autoimmune, inflammatory, cardiovascular and nervous system disorders), as well as in cancer [37,38,39,41]. Moreover, constitutive EGF system activation and uncontrolled ErbB signaling may disrupt the balance in cell cycle regulation (proliferation, differentiation, migration and apoptosis), sensitize cells to oncogenic transformation and trigger ErbB-induced oncogenesis [37,38,39,41,42].

More specifically, in malignant transformation, the EGF system becomes hyperactivated with the following four main mechanisms: gain of function mutations, genomic amplification, chromosomal rearrangements and autocrine activation [40,41,42].

### 5.1. Gain of Function Mutations

The gain of function (GOF) mutations may have a crucial role in carcinogenesis, as they generate novel protein isoforms with new and important functions [125]. Based on their consequences for cancer development, GOF mutations could be further subclassified into driver and passenger mutations [125,126]. Driver mutations provide a selective cell growth advantage and promote cancer development, while passenger mutations do not confer any cell growth advantage and do not contribute to carcinogenesis [126,127].

Especially in the EGF system, GOF mutations could possibly affect most domains of an ErbB receptor and lead to aberrant downstream signaling [42]. More specifically, a GOF mutation usually involves the bilobed tyrosine kinase domain of an ErbB receptor and causes tyrosine kinase hyperactivation and aberrant downstream signaling, as well as conferring oncogenic properties [42]. However, GOF mutations could also affect various ErbB receptor domains (the extracellular ligand-binding domain, transmembrane domain and short juxtamembrane section) and cause receptor activation using alternative mechanisms [42].

### 5.2. Genomic Amplification

Genomic amplification is the copy number increase in a specific region of the genome and is associated with overexpression of the amplified genes [128,129]. It usually occurs during development and carcinogenesis and may be promoted by common chromosomal fragile sites, errors in DNA replication or telomere dysfunction [129,130]. Amplified sequences can be organized as extrachromosomal elements, repeated units at a single locus or interspersed throughout the genome [128,129].

Especially in the EGF system, genomic amplification and subsequent ErbB receptor overexpression leads to increased receptor local concentration, constitutive receptor activation, avoidance of receptor regulatory mechanisms and aberrant downstream signaling [42,131,132]. More specifically, ErbB-2 overexpression causes constitutive ErbB-2 activation as well as EGFR ligand-independent activation [131]. Moreover, ErbB-2 overexpression inhibits down-regulation mechanisms of ErbB-2 and EGFR [131].

### 5.3. Chromosomal Rearrangements

Chromosomal rearrangements have important roles in carcinogenesis and include deletions, duplications, inversions and translocations [133]. They are mainly caused by either defective DNA double strand break repair or faulty DNA replication [134]. Based on their effect on chromosomes, they could be further subclassified into simple and complex [134]. Simple chromosomal rearrangement results from a single fusion that preserves genetic information but sometimes disrupts regulation of the genes involved [134]. In contrast, complex chromosomal rearrangement results from multiple fusions at a single locus that cause changes in genetic content and in chromosomal linear structure [134]. Overall, chromosomal rearrangements lead to either hybrid gene formation or gene dysregulation [133,134].

Especially in the EGF system, chromosomal rearrangements cause the formation of fusion oncoproteins, consisting partly of the ErbB receptor and partly of the fusion partner [42,135]. These fusion oncoproteins have remarkable structural similarities, can be membrane bound or cytoplasmic, and contain an activated tyrosine kinase domain [42,135].

### 5.4. Autocrine Activation

Autocrine activation is a type of self-stimulation in which a cell secretes a hormone-like factor that binds functional receptors on the same cell [136]. This type of cell signaling has a significant role in carcinogenesis, particularly in cases of constitutive autocrine activation [136,137,138].

Especially in the EGF system, autocrine activation of ErbB receptors is a well described phenomenon that leads to downstream signaling via several pathways and may confer oncogenic properties [42,139,140].

## 6. ErbB Receptors in Endometrial Cancer

During the menstrual cycle, there is a wide variation in the profile of ErbB receptors, indicating a central role of the EGF system in the regulation of endometrial cyclical growth and shedding [141,142].

In EC, the expression of ErbB receptors is significantly different, compared with the premenopausal and postmenopausal endometrium [141,143,144]. This is mainly because of the increased transcriptional activity of ErbB encoding genes in EC cells [144].

### 6.1. Profile of ErbB Receptors in Endometrial Cancer

Overall, EGFR overexpression is reported in 43–67% of unselected EC cases [144,145,146,147,148,149,150,151,152,153,154,155]. EGFR overexpression is present in approximately 46% of type I EC (endometrioid) cases [149,151,156]. EGFR overexpression is observed in 34–50% of type II EC (papillary serous, clear cell, undifferentiated) cases [149,151,156,157,158,159].

ErbB-2 overexpression and ΕrbB-2 gene amplification represents a very rare event in unselected EC cases [144,149,153,154,155]. However, ΕrbB-2 overexpression and ΕrbB-2 gene amplification are present in only 8–15% and 3% of type I EC cases, respectively [144,149,156,160,161,162,163]. In contrast, ΕrbB-2 overexpression and ErbB-2 gene amplification are more common in type II EC cases [149,151,156,157,158,159].

Moreover, the exact frequency of ErbB-2 overexpression and ΕrbB-2 gene amplification in type II EC remains controversial, as there are many racial differences [149,151,157,164,165]. More specifically, ErbB-2 overexpression and ΕrbB-2 gene amplification are more common in African—American patients with type II EC, when compared with Caucasian individuals [164,165].

Likewise, ErbB-2 overexpression and ΕrbB-2 gene amplification have significant variations among different histologic subtypes of type II EC [149,151,157,160,165,166,167]. ErbB-2 overexpression and ΕrbB-2 gene amplification are reported in 18–80% and 17–47% of papillary serous EC cases, respectively [149,151,160,163,165,166,167], and 33% and 16–50% of clear cell EC cases, respectively [149,151,160,167].

ErbB-3 overexpression is reported in 30% of unselected EC cases [141,153]. More specifically, ErbB-3 overexpression is more common in well differentiated tumors when compared with moderately and poorly differentiated ones [141].

Similarly, ErbB-4 overexpression is reported in 15% of unselected EC cases [141,153].

Overall, there are some differences in ErbB-2 receptor profile in selected EC patients (EC histologic subtypes and racial—ethnic subgroups) [143,150,157,164]. ErbB-2 receptor expression is more common in papillary serous and clear cell EC cases [143,150,157]. This is mainly based on differences in the pathophysiology and clinical behavior of various EC histologic subtypes [143,150,157].

### 6.2. Clinical Role in Endometrial Cancer

The relationship of the ErbB receptors profile with disease stage, tumor grade and response to treatment remains controversial in EC cases [149,153].

In particular, the clinical role of EGFR overexpression has not been studied thoroughly in EC patients [149,153]. Some studies demonstrate an association between EGFR overexpression and poor clinical outcome, while others report otherwise [145,146,147,148]. It seems that EGFR overexpression may have a dual role in EC cases [149]. EGFR overexpression in type I EC is associated with less aggressive disease and more favorable outcomes [149,151,153,157]. In contrast, EGFR overexpression in type II EC is associated with more aggressive disease and adverse clinical outcomes [149,151,153,157].

However, the clinical significance of ΕrbB-2 overexpression and ΕrbB-2 gene amplification has been studied extensively in EC patients [151,157,160,165,166,168,169,170]. ΕrbB-2 overexpression and ΕrbB-2 gene amplification are indicators of a more aggressive disease with reduced response to treatment and less favorable outcomes, especially in patients with type II EC [151,153,157,160,165,166,168,169,170,171].

Furthermore, the clinical role of ErbB-3 and ErbB-4 overexpression has not been studied extensively in patients with EC [141,143,150,151,152,153,157].

It becomes apparent that ErbB-2 receptor expression is more common in aggressive EC histologic subtypes (papillary serous and clear cell) [143,150,157]. This possibly indicates a future role of ErbB-targeted therapies in well-defined EC subgroups with overexpression of ErbB receptors [150,157,172].

## 7. Conclusions

Overall, the EGF system signaling network becomes hyperactivated with various mechanisms and possibly participates in EC pathogenesis via several signaling pathways [37,38,39,40,41,42]. There are some differences in ErbB-2 receptor profile among EC subgroups that could be explained by the differences in pathophysiology and clinical behavior of various EC histologic subtypes [143,150,157].

The fact that ErbB-2 receptor expression is more common in aggressive EC histologic subtypes (papillary serous and clear cell), might indicate a future role of ErbB-targeted therapies in well-defined EC subgroups with overexpression of ErbB receptors [150,157,172]. In this context, future studies are needed in order to evaluate thoroughly the effectiveness of ErbB-targeted therapies as single agents or adjuvant treatment in well-defined EC subgroups with overexpression of ErbB receptors [7,21,172,173,174,175,176,177,178].

## Figures and Tables

**Table 1:** ErbB ligands and their affinity for ErbB receptors.

	ErbB-1	ErbB-2	ErbB-3	ErbB-4
EGF	+	-	-	-
TGF-a	+	-	-	-
Amphiregulin	+	-	-	-
HB-EGF	+	-	-	+
Betacellulin	+	-	-	+
Epigen	+	-	-	+
Epiregulin	+	-	-	+
Neuregulin-1	-	-	+	+
Neuregulin-2	-	-	+	+
Neuregulin-3	-	-	-	+
Neuregulin-4	-	-	-	+
Neuroglycan C	-	-	+	-
Tomoregulin	-	-	-	+

+ means positive, while - means negative.
